# Ribonucleotide reductase subunit M1 blocks glucose catabolism via phosphorylation of pyruvate kinase M2 in human esophageal squamous cell cancer

**DOI:** 10.1016/j.gendis.2023.04.031

**Published:** 2023-06-24

**Authors:** Jian Yang, Lingxiao Wang, Hui Sun, Zhenxiang Zhao, Yanchuan Ma, Zhen Hu, Liqin Zhai, Yonggang Wang

**Affiliations:** a Translational Medicine Research Center, Shanxi Medical University, Taiyuan, Shanxi 030001, China; b Department of Cell Biology and Genetics, Shanxi Medical University, Taiyuan, Shanxi 030001, China; c Key Laboratory of Cellular Physiology of the Ministry of Education, Shanxi Medical University, Taiyuan, Shanxi 030001, China; d Department of Colorectal Surgery, The Fifth Clinical Medical College of Shanxi Medical University, Taiyuan, Shanxi 030012, China; e Science & Technology Information and Strategy Research Center of Shanxi, Taiyuan, Shanxi 030006, China; f Department of Pathology, Shanxi Provincial People's Hospital, Taiyuan, Shanxi 030012, China; g Department of Anatomy, College of Basic Medicine, Shanxi Medical University, Taiyuan, Shanxi 030001, China

Esophageal squamous cell cancer (ESCC) is one of the malignant tumors with high morbidity and mortality all over the world.^1^ In recent years, combined chemotherapy has gradually been the effective treatment method for ESCC patients. Therefore, it is imperative to explore potential therapeutic targets for the treatment of ESCC. We previously reported a high-frequency amplification of pyrimidine metabolic pathway-related genes in ESCC tissues,^2^ and ribonucleotide reductase subunit M1 (RRM1) was one of many abnormal genes. RRM1 is one of the important subunits of ribonucleoside reductase (RR) and plays a key role in cell DNA replication as a binding site of nucleotides to regulate substrate specificity and RR activity. RRM1 is expressed in almost all tumor cells, but its expression levels and functions are differently affected by tissue origin and cell location. The expression of RRM1 in lung cancer tissues was generally lower than that in adjacent tissues, while an opposite trend was observed in breast cancer. Reports about RRM1 in ESCC are less, and the mechanism(s) of carcinogenesis is still poorly understood. The present study aimed to understand the role of RRM1 on the malignant proliferation process, and further clarify the molecular mechanism in ESCC development, which would be helpful in anti-cancer therapy of ESCC patients.

Data obtained from the GEPIA database and immunohistochemical results showed that RRM1 expression in most ESCC tissues was abnormally higher than that in adjacent tissues (Fig. S1; Fig. 1A, B), and a significantly negative correlation between the RRM1 expression and survival time was observed, and patients with high RRM1 expression exhibited a worse prognosis (Fig. 1C), suggesting that RRM1 could be used as an indicator for early diagnosis, a potential predictor to estimate patients' survival or a potential target of antitumor drugs. RRM1 is often considered as a tumor suppressor gene inhibiting tumor cell proliferation, metastasis, and invasion, and is used as the main target of antitumor drugs. However, clinical trials showed the high sensitivity of lung cancer patients with low RRM1 expression to gemcitabine, and the chemotherapy scheme with gemcitabine is often used to treat the patients with low RRM1.^3^ The response to clinical chemotherapy targeting RRM1 is closely related to the expression levels of RRM1 in cancer tissue. Different sensitivity of RRM1 on antitumor drugs was close to the carcinogenic mechanism of RRM1, but the mechanism has remained unclear.

**Figure 1:**
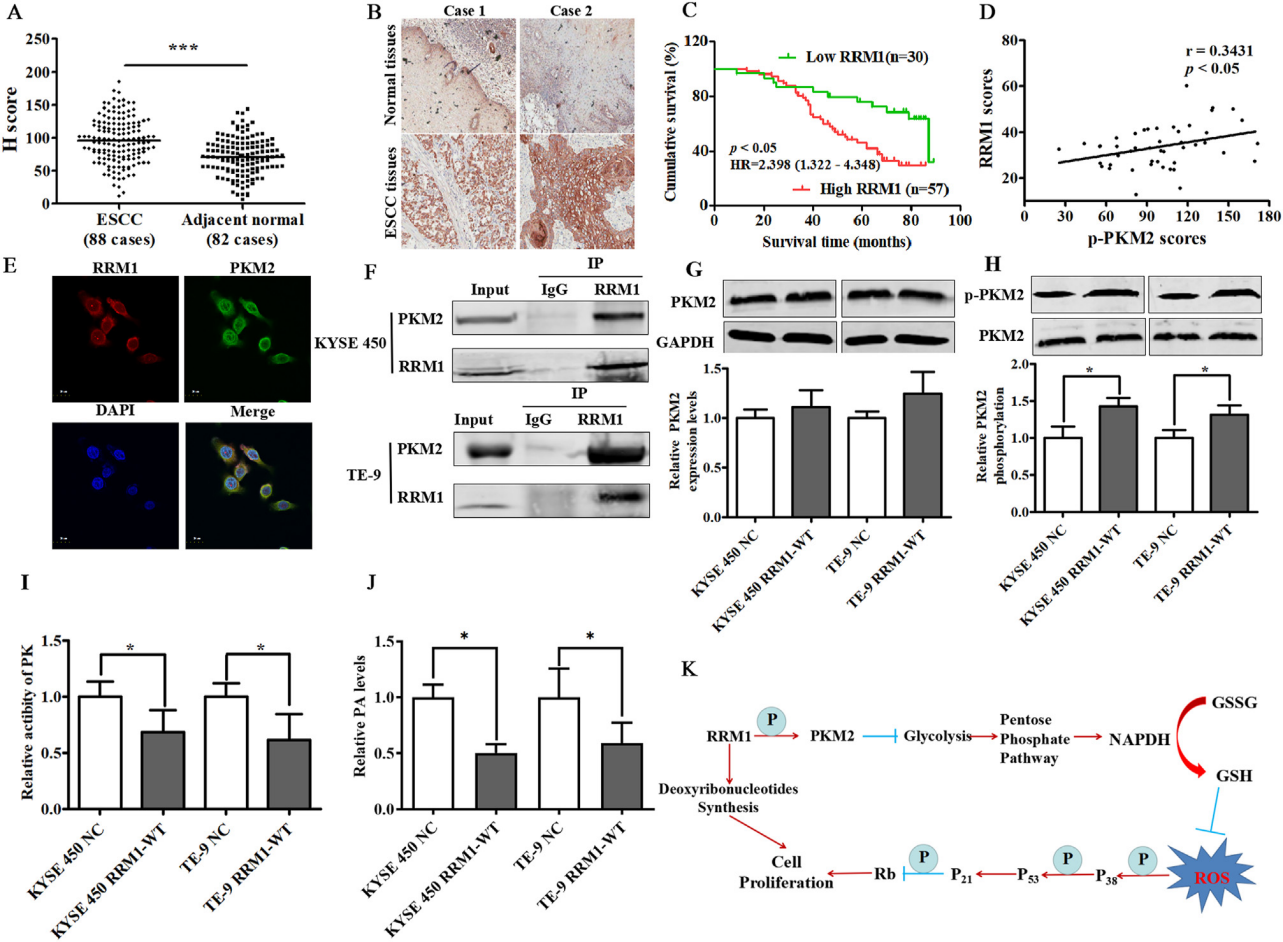
RRM1 induces tumorigenesis via phosphorylation of pyruvate kinase M2 in human esophageal squamous cell cancer (ESCC). **(A)** Histochemical scoring of RRM1 expression in ESCC and matched paracancerous tissue samples (∗*P <* 0.05). **(B)** Representative images of RRM1 protein expression in ESCC tissue and normal tissue vis immunohistochemical analysis (Scale bar = 100 μm). **(C)** Kaplan–Meier survival analysis of overall survival of ESCC patients. **(D)** The correlation analysis between RRM1 and p-PKM2 expression was carried out by Pearson correlation coefficient. **(E)** Expression of RRM1 (red) and PKM2 (green) in cells was detected by immunofluorescence. **(F)** The interaction between RRM1 and PKM2 was confirmed by IP experiment. **(G, H)** PKM2 and phosphorylated PKM2 (p-PKM2) were detected by Western blot assays. **(I, J)** PK activities and PA levels were shown by bar graphs. **(K)** Regulation model of RRM1 on cell proliferation in ESCC. Arrows (→) and truncated lines (—**|**) indicated promoting and inhibiting effects, respectively. Data were representative of three independent experiments and are presented as mean ± standard deviation, and *P* values < 0.05 were considered statistically significant.

To study the regulatory mechanism of RRM1, we first verified its biological effect in human ESCC cells. By siRNA interfering with RRM1 in ESCC cell lines, it was found that the S phase of the cell cycle was significantly increased, but the G2 phase was significantly decreased, indicating that the cell cycle was blocked in the S/G2 phase so that cell proliferation was inhibited (Fig. S2, 3). Results from RRM1-overexpressing ESCC cells further proved the key role of RRM1 on the cell cycle. Intracellular up-regulated RRM1 expression promoted the mass production of DNA synthetic substances providing material support for cell division, which were confirmed by targeted metabonomics analysis and HPLC-electrochemical detection identified more 2′-Deoxyguanosine 5′-monophosphate (dGMP), deoxycytidine monophosphate (dAMP), deoxyadenosine monophosphate (dCMP), and deoxyinosine in RRM1-overexpressing cells (Fig. S4). The nucleic acid metabolism is closely related to the pentose phosphate pathway (PPP),^4^ and the increased d-Ribose 5-phosphate (R5P) as substrates of nucleic acid synthesis from PPP indicated that the up-regulation of nucleic acid anabolism may be partly promoted by the enhancement of PPP. Moreover, the up-regulation of intermediate metabolites of glycolysis, including d-glucose 6-phosphate (G6P), phosphoenolpyruvate (PEP), reduced nicotinamide adenine dinucleotide (NADH), and adenosine triphosphate (ATP), was observed, but the levels of l-lactic acid and malic acid involved in the tricarboxylic acid cycle were down-regulated. These indicated that glucose catabolism in RRM1-overexpressing cells might be blocked, which could promote more carbon flowing into PPP and nucleic acid anabolism.

The enhanced PPP not only promotes nucleic acid anabolism, but also significantly increases the intracellular co-enzyme II, and the up-regulated reduced nicotinamide adenine dinucleotide phosphate (NADPH) and nicotinamide adenine dinucleotide phosphate (NADP^+^) in RRM1-overexpressing cells was observed. NADPH is an important intermediate metabolite in PPP and provides a necessary co-factor that plays an essential role in lipogenesis and the entire antioxidant system. In the defense against intracellular oxidative stress, one of the major antioxidants is glutathione (GSH) which neutralizes reactive oxygen species by the synthesis of oxidized glutathione disulfide (GSSG), and the cycling of GSSG to GSH depends on NADPH. The detection of GSH and GSSG confirmed the enhanced glutathione system and relieved oxidative stress (Fig. S5). The reactive oxygen species (ROS) levels in cells, unexpectedly, were down-regulated. Lower ROS down-regulated three key tumor suppressor proteins (p-p38, p-p53, and p21; Fig. S5F), which up-regulated Rb phosphorylation and promoted cell cycles. Taken together, the enhanced glutathione system was promoted by a large number of NADPH provided from PPP, and relieved oxidative stress promoted cell proliferation of human ESCC by activating cell division via ROS-mediated p-p38/p-p53/p21/p-Rb pathway.

Undoubtedly, the blocked glucose catabolism may directly or indirectly result from RRM1. To explore the potential regulation mechanism, protein mass spectrographic analysis was performed. Pyruvate kinase M2 (PKM2) was found to be the potential protein that interacted with RRM1, and the interaction relationship between both proteins was confirmed by immunocoprecipitation (Fig. 1E, F). In the glycolytic pathway, pyruvate kinase (PK) is a key enzyme that catalyzes the transfer of a phosphoryl group from phosphoenolpyruvate to ADP to form pyruvic acid (PA) and ATP, and the PK activity is closely related to the phosphorylation of PKM2.^5^ In the present study, the up-regulated phosphorylation levels of PKM2 were observed in both RRM1-overexpressing cells (Fig. 1G, H), and the inhibited PK activity and reduced PA were also found correspondingly (Fig. 1I, J), indicating that hyperphosphorylation of PKM2 might be the main reason causing the decreased PK activity and impaired glucose decomposition. The immunohistochemical analysis further revealed a significant positive correlation between the expression levels of RRM1 and p-PKM2 (Fig. 1D).

Carcinogenesis of RRM1 may work by controlling the phosphorylation of PKM2 in human ESCC. To further clarify the regulatory mechanism, an inhibitor (gemcitabine) of RRM1 activity was used in RRM1-overexpressing cells. Results showed that the PKM2 phosphorylation level significantly decreased compared to that of control groups (Fig. S6A), but the PK activity and PA levels significantly increased (Fig. S6B, C). The enhanced PK activity may promote more intermediates into the tricarboxylic acid cycle, and reduce the carbon flux to PPP, inhibiting the synthesis of antioxidants and re-increasing the oxidative stress levels (Fig. S6D). Increased oxidative stress resulted in slowed cell cycle progression, and less deoxyribonucleotide caused by reduced RRM1 activity inhibited DNA production, finally slowing down cancer cell proliferation (Fig. S6E). Similar results have been achieved by the applied activator of PKM2 activity (TEPP-46) in RRM1-overexpressing cells, indicating a better clinical efficacy in the treatment of gemcitabine and TEPP-46 on ESCC.

The present study has identified RRM1 as a cancer-promoting gene by promoting cell cycle progression in human ESCC. We propose a potential prevention strategy of RRM1 through shunting glucose-derived carbon between glucose catabolism and pentose phosphate pathway and reveal the blocking mechanism of RRM1 on glucose catabolism works by a phosphorylation-independent mechanism on PKM2 as shown in Fig. 1K. The multifaceted role of RRM1 in cell metabolism and cell cycle progression makes it a valuable target to improve the clinical efficacy of ESCC chemotherapy.

## Author contributions

J.Y., L.W., and H.S. developed the ideas and drafted the manuscript. J.Y., L.W., H.S, Z.Z., and Y.M. conducted the experiments. Z.H., L.Z., and Y.W. contributed to the data analysis. J.Y. revised the manuscript. All authors read and approved the final version of the manuscript for publication.

## Conflict of interests

All authors declare that they have no conflict of interests.

## Funding

This research was supported by the 10.13039/501100001809National Natural Science Foundation of China (No. 82103175), and the 10.13039/501100004480Natural Science Foundation of Shanxi Province, China (No. 201901D111432, 20210302123316, 202103021224379).

## Figures and Tables

**Fig. S1:**
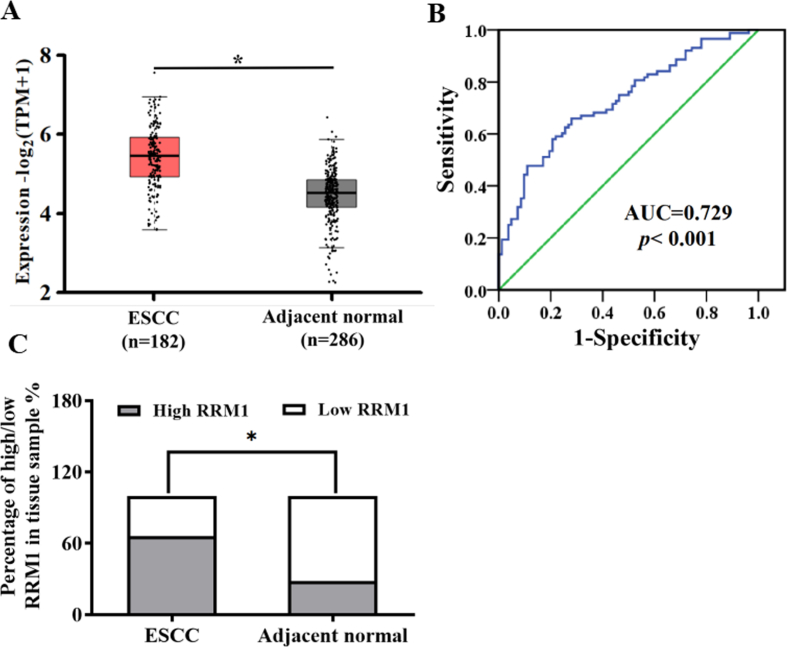
RRM1 expression analysis in human ESCC clinical samples. **(A)** Comparative analysis of RRM1 mRNA expression in ESCA tissues (*n* = 182) and normal tissues (*n* = 286) from the GEPIA online database. **(B)** Receiver operating characteristic (ROC) curve for RRM1 protein expression in CRC tissues and normal tissues. **(C)** The proportion of high and low RRM1 protein expression in ESCC and normal tissue samples based on the cutoff value from ROC curves.

**Fig. S2:**
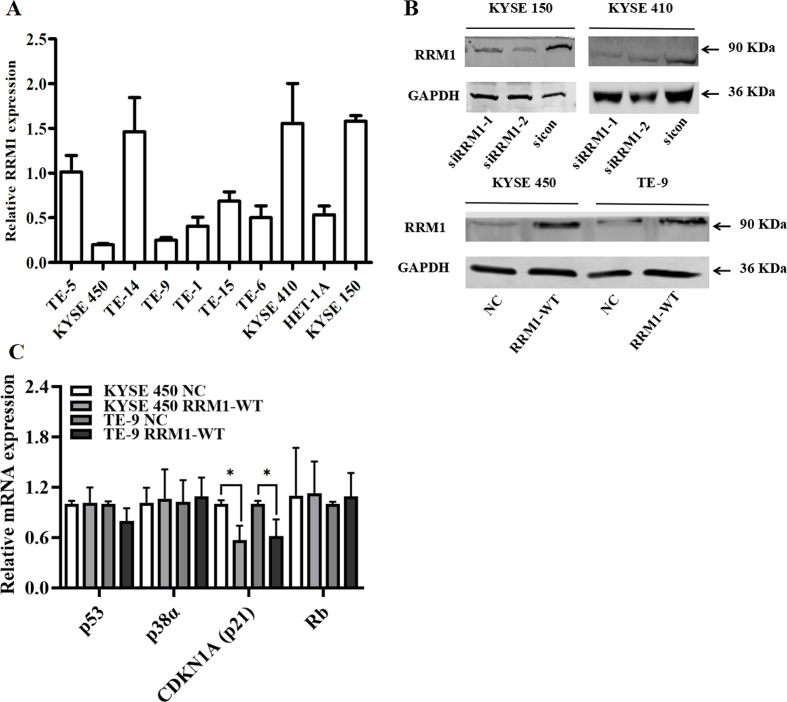
Detection of genes or protein expression in different ESCC cell lines. **(A)** Levels of RRM1 expression in ESCC cell lines and immortalized esophageal epithelial cells detected by real-time quantitative PCR. **(B)** Effects of RRM1 knockdown in KYSE150 and KYSE410 or its overexpression in KYSE50 and TE-9 was determined by western blot. **(C)** Relative expression levels of p53, p38α, CDKN1A (p21), and Rb mRNA in RRM1-overexpressing cells were detected by qRT-PCR.

**Fig. S3:**
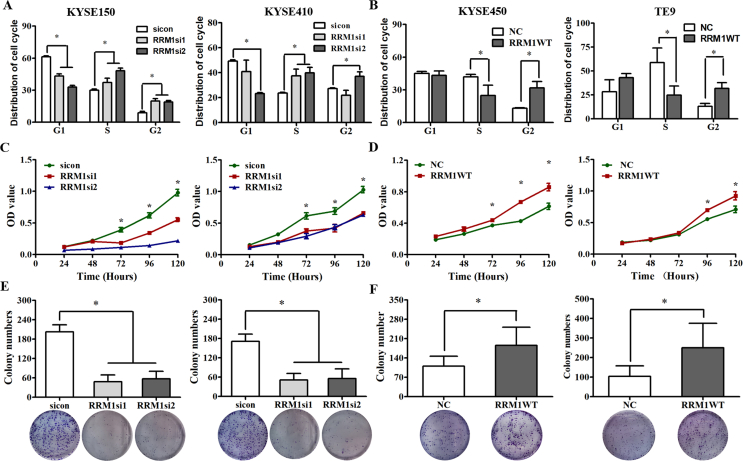
RRM1 induces tumorigenesis. **(A, B)** Changes in each stage of the cell cycle in RRM1-knockdown cells (KYSE150 and KYSE410), and RRM1-overexpressing cells (KYSE50 and TE-9) were observed by flow cytometry. **(C, D)** Cell proliferation assays were performed in RRM1-knockdown cells, RRM1-overexpressing cells, and corresponding control cells, respectively. **(E, F)** Colony formation assays were performed in RRM1-knockdown cells, RRM1-overexpressing cells, and corresponding control cells, respectively. Data were representative of three independent experiments and are presented as mean ± standard deviation, and *P* values < 0.05 were considered statistically significant.

**Fig. S4:**
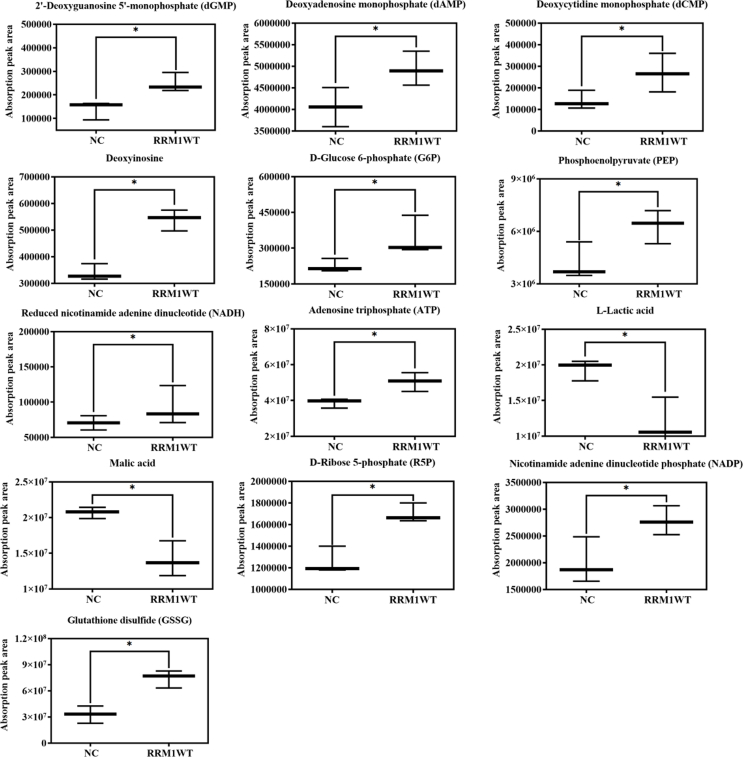
Targeted metabonomics analysis of RRM1-overexpressing cells as the treatment group and cell lines with an empty plasmid as the control group. Three repetitions were performed, and all data were expressed as mean ± standard deviation, and *P* values < 0.05 were considered statistically significant.

**Fig. S5:**
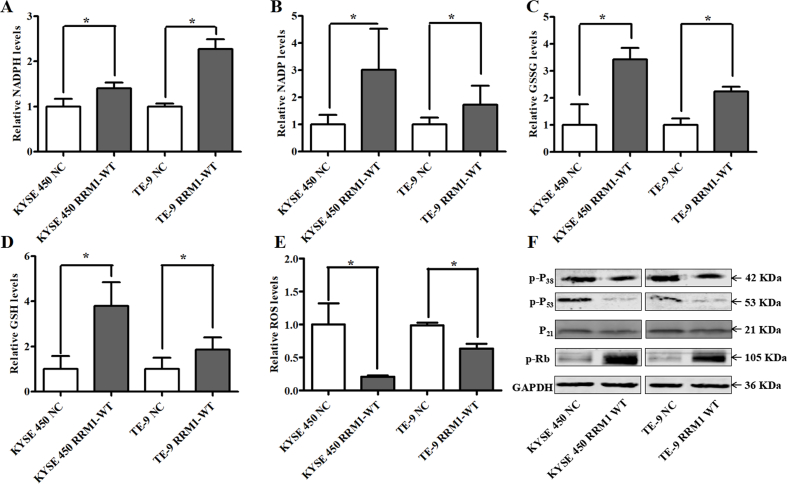
RRM1 reduces intracellular oxidative stress by enhancing antioxidative activities and induces cell division through the ROS-mediated p38/p53/p21/p-Rb pathway. Levels of NADP **(A)**, NADPH **(B)**, GSSG **(C)**, GSH **(D)**, and ROS **(E)** in cell lines. **(F)** The levels of Phospho-p38 (p-p38), phospho-p53 (p-p53), p21, and phospho-Rb (p-Rb) were examined in both RRM1-overexpressing cell lines, respectively. Data were representative of three independent experiments and are presented as mean ± standard deviation, and *P* values < 0.05 were considered statistically significant.

**Fig. S6:**
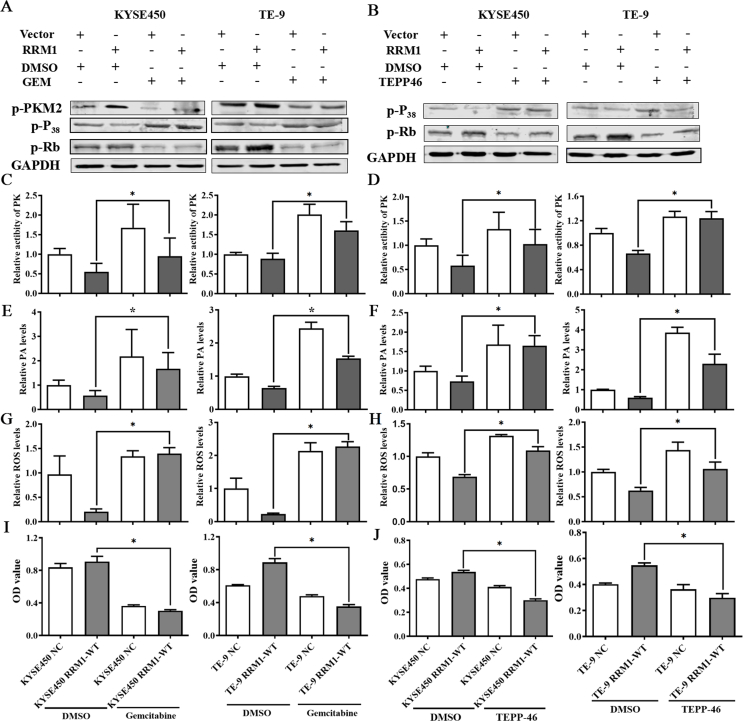
Analysis of cell viability and oxidative stress after drug treatment. **(A)** Gemcitabine inhibited the expression of p-PKM2 and p-Rb, up-regulated p-p38 levels, reactivated the PK activity **(C)**, and increased PA levels **(E)** in RRM1-overexpressing cells. **(B)** TEPP-46 resulted in the up-regulated p-p38 expression **(H)**, PK activity **(D),** and PA levels **(F)**, but down-regulated the expression of p-Rb. **(G, H)** Levels of ROS in cell lines with drugs. MTT assays were performed after treatment with gemcitabine (20 μM, 48 h) **(I)** and TEPP-46 (200 μM, 48 h) **(J)** in the RRM1-overexpressing cells and corresponding control cells. Data were representative of three independent experiments and are presented as mean ± standard deviation, and *P* values < 0.05 were considered statistically significant.
