# Supplementary material for: Ribonucleotide reductase subunit M1 blocks glucose catabolism via phosphorylation of pyruvate kinase M2 in human esophageal squamous cell cancer
Source: Genes Dis. 2023 Jun 24;11(3):101000. doi: 10.1016/j.gendis.2023.04.031 (PMC10808956; doi:10.1016/j.gendis.2023.04.031)
Supplement: Multimedia component 1 [file mmc1.docx]

**Materials and methods**

**RRM1 expression analysis in human clinical samples**

Ethical approval in present study was provided by the Human Ethics Committee of Shanxi Medical University (2019LL131). 105 cases paraffin-embedded ESCC tumor samples were retrieved from the department of pathology in Shanxi provincial people's Hospital, and written informed consent from each patient was obtained after a detailed explanation. The clinicopathological characteristics of ESCC patients could be found in Additional files (Tab. S1). This study was performed in accordance with the standards of the Declaration of Helsink. The RRM1 expression in samples was detected by human tissue microarray technology (TMA) and immunohistochemistry. Finally, the images and data are collected using the ScanScope slide scanning system (Aperio Technologies Inc., Vista, CA, USA).

**“Insert Tab. S1”**

**Cell culture, RRM1 silencing and overexpression in ESCC cells**

Human esophageal epithelial cell lines (KYSE150、TE-1、KYSE450 and TE-9) used in this study were obtained form the Key Laboratory of Cellular Physiology and Translational Medical Research Center, Shanxi Medical University (Taiyuan, Shanxi, China). All cell lines were cultured in Dulbecco's modified Eagle medium (Invitrogen, Carlsbad, CA, USA) containing 10% calf serum at 37 ℃ in 5% CO_2_. Knockdown of endogenous RRM1 was carried out by RRM1-targeted short interfering RNA (siRRM1). Two siRRM (5′-GTGGAATGTTACTCCTACA-3′named as siRRM1-1, 5′-GCACAGAAATAGTGGAGTA-3′ named as siRRM1-2) and a negative siRNA (5′- TTCTCCGAACGTGTCACGTTTC-3′ named as sicon) was purchased from Guangzhou RiboBio Co., Ltd (Guangzhou, China).

Human RRM1 cDNA was purchased form PolePolar Biotehnology Co., LTD (Beijing, China) and cloned into the pCMV-HA vector. Transfection with all cDNA constructs produced the predicted protein product (named as RRM1-WT), and the matching empty vectors were used as negative control (named as NC). The efficiency of RRM1 silencing and overexpression was determined via Western blot.

**Cell proliferation and Cell cycle analyses**

Cell proliferation was examined by MTT assay and soft-agar colony forming assay. MTT assay: The cells were seeded in 96 well plates at a density of 4000 cells per well. After 24 h, 48 h, 72 h and 96 h of culture, 20 µL MTT (5 mg/ml in DMEM) was added to each well. After 4 hours of incubation, dimethyl sulphoxide (DMSO) was added. After 10 minutes, the absorbance at 490 nm of each well was measured by Multimode Reader (Varioskan Flash, Thermo Electron Co. US). Soft agar colony forming assay: Each agarose disk was inoculated with 1000 cells, and added with cell culture medium. After 12 days. Cells was fixed with 5% trioxane, dyed with crystal violet. washed with PBS, and the number of colony forming was counted.

Cell cycle analysis was performed by PI staining and flow cytometry (FAGS). Cells were collected and fixed in 75% ethanol at 4℃. Cells were then were resuspended with 1 ml PI/Triton X-100 staining solution of 0.2 mg RNase A and stained at 37 ℃ for 15 min and analysed by FAGS.

**qRT-PCR analysis.**

Using MMLV reverse transcriptase (TaKaRa, Takara Shuzo Co. Ltd., Japan), reverse transcription was performed, then the expression of gene mRNA was determined using the SYBR® Green PCR Master Mix (Vazyme Biotech Co., Ltd) by ABI 7500 Real-Time PCR System (Applied Biosystems, Foster City, CA, USA). The qRT-PCR primers were shown in Table S2, and qRT-PCR reaction mixture was shown below: 0.4 μL each forward and reverse primers (10 μM), 1 μL cDNA template, 10 μL SYBR real-time PCR premixture (2×), and RNase free dH_2_O to adjust to 20 μL. Using the 2^-ΔΔCt^ method (17), relative quantification of gene mRNA expression was calculated.

**“Insert Table S2”**

**Western blotting**

Cultured cells were lysed in RIPA buffer (50 mM Tris/HCl, pH 7.4, 150 mM NaCl, 0.25% deoxycholic acid, 1% NP-40, 1 mM EDTA) with protease and phosphatase inhibitor mixtures (Thermo) at 4℃. After 1 h, lysates were centrifuged at 10,000 g at 4℃ for 30 min, and the concentrations of protein in supernatant were determined. Equal amounts of proteins were separated by 10% SDS-PAGE, and then were separated and transferred onto PVDF membranes (Whatman Plc, Maidstone, Kent, UK) incubated with PBST buffer (pH 8.0) containing 5% nonfat dry milk. After washing three times in PBST, the separated proteins were probed with the special antibodies including anti-RRM1 antibody (ab137114, Abcam), anti-p38 (phosphoT180, ab178867), anti-p53 (phospho S15, ab1431), anti-p21 (ab109520), phospho-S807-Rb (8180T, Cell Signaling Technology), PKM2 (15822-1-AP, Proteintech), anti-PKM2 (phospho S37, PA5-37684, Invitrogen) by incubating overnight at 4 ℃. The blot was detected with IRDye 680RD goat anti-rabbit/mouse antibodies (925-68071/68080, Li-Cor Biosciences), and was detected with LI-COR Odyssey Imaging System (LI-COR Biosciences, Bad Homburg, Germany). GAPDH (Proteintech Group, Wuhan, China) was used as loading control.

**Metabolomic profiling with Liquid Chromatograph Mass Spectrometer (LC-MS)**

Metabolomic profiles of cells with RRM1 overexpression and corresponding control samples were achieved using LC-MS platform (Shanghai applied protein technology co.ltd, China). 2×10^7^ cells were collected and added with 1ml organic mixture (methanol:acetonitrile:water = 2:2:1, V/V). The mixture was vortexed for 1 min and centrifuged (13000rpm, 10 min, 4 ℃ ), and the supernatants were collected and evaporated to dryness. After that the dried samples were redissolved in 100ul acetonitrile aqueous solution (acetonitrile: water = 1:1, V / V) and centrifuged at 14000g for 10 min at 4 ℃ , a 2-μl supernatants were injected into the Agilent 1260 apparatus coupled with the Agilent 6460 Triple Quadruple mass spectrometer in the multiple reaction monitoring mode. For LC, the mobile phase A was H_2_O plus 25mM ammonium acetate and 25 mM ammonia (pH 9.75), and the mobile phase B was acetonitrile. After the pretreatment of baseline correction, de-noising, smoothing, alignment, time-window splitting, and multivariate curve resolution, raw data containing retention time, intensity, and the mass-to-charge ratio of each peak were obtained. The intensity data of these metabolites were interpreted using the MRM Analyzer.

**Detection of glutathione, pyruvate kinase activity, pyruvic acid level, and oxidative stress**

Intracellular GSH and GSSG contents, pyruvate kinase enzymatic activity, and pyruvic acid levels were measured using a commercially available kit (S0053, eyotime Institute of Biotechnology, Shanghai, China), pyruvate kinase activity spectrophotometry assay kit (BC0540, Solarbio, Beijing, China), and pyruvic acid assay kit (BC2200, Solarbio, Beijing, China) following the manufacturer's instructions, respectively. Reactive oxygen species was detected by chemical fluorescence method using a reactive oxygen species assay kit (E004-1-1, Nanjing Jiancheng Bioengineering Institute, China). Dichloro-dihydro-fluorescein diacetate (DCFH-DA, 10 µM) was added to cells in darkness. After 15 min incubation at 37 ℃, cells were harvested and washed twice with PBS buffer at 4 ℃, and was suspended in PBS buffer for the fluorescence detection using a flow cytometer.

**Immunocoprecipitation**

Cells were collected and lysed in lysis buffer containing a phosphatase inhibitor (cocktail, Sigma) for co-immunoprecipitation analyses. 500 ul protein lysate was used for immunoprecipitation with 4ug anti-RRM1 antibody (ab137114, Abcam) or anti-PKM2 antibody (15822-1-AP, Proteintech) overnight at 4℃, and was followed by incubation with Protein A/G PLUS-Agarose (from Santa Cruz) conjugated to Magnetic beads for 30 minutes. Beads were collected and washed with precooled lysis buffer for 5 times. The proteins were resolved on SDS–PAGE, and transferred to nitrocellulose membranes (GE Healthcare Life Sciences) and probed with anti-RRM1 or anti-PKM2 antibodies. The same amount of normal rabbit IgG-AC (sc-2345, Santa Cruz) was used as a control.

**Drug treatment**

Gemcitabine (HY-17026 ) and TEPP-46 (HY-18657) was purchased from MedChem Express (MCE, USA). Stock solutions of 10 mM (Gemcitabine) and 10 mM (TEPP-46) were prepared in DMSO. All dilutions to working solution were performed in appropriate cell culture media. In the experiments of measuring the intracellular PK activity, phosphorylation of endogenous PKM2, and intracellular ROS levels, cells were treated with 20µM Gemcitabine or 200µM TEPP-46 for 48h.

**Statistics analysis**

Data were presented with mean ± standard deviation (S.D.) from three independent experiments. All statistical analysis was performed using SPSS software (version 17.0, SPSS Inc., Chicago, IL, USA), and data graphs were generated using GraphPad Prism 5 Software (GraphPad Software Inc. La Jolla, CA, USA). The correlation analysis between RRM1 expression with clinical information and Phos-PKM2 were carried out using Chi square test and Pearson correlation coefficients, respectively. The receiver operating characteristic curve was used to determine cutoff value between high expression and low expression of RRM1 protein in ESCC tissues. Survival curves were constructed using the Kapla-Meier method and differences in survival were evaluated using the log-rank test. *p* values were performed using Student’s t-test and one-way ANOVA analysis between groups, and a value of *p* < 0.05 was considered as statistically significant which was denoted with *.

**Results:**

**Up-regulated RRM1 expression in ESCC tissues**

Data obtained from the GEPIA database found an elevated expression of RRM1 mRNA in ESCC tissues (Fig. S1A), and the imunohistochemical results also showed that the expression of RRM1 protein in malignancy tissues was significantly higher than that in contiguous tissues (H score values: 95.90 ± 2.82 vs 71.22 ± 2.44, P<0.0001, Fig. 1A, B). Logistic regression model and receiver operating characteristic (AUC = 0.729, P < 0.001; Fig. S1B) curves were carried out to evaluate the optimal cutoff value (91.91) and divide all tissue samples into low-RRM1 expression group and hight-RRM1 expression group. Rank sum test showed a significant differences of distribution between the different groups in ESCC tissues and normal tissues. RRM1 protein expression was generally high in ESCC tissues (Fig. S1C). Chi-square test found that high RRM1 expression was statistically associated with T classification (Tab.S3). Univariate and multivariate Cox regression analyses showed that RRM1 expression and AJCC stage was significantly associated with overall survival (Tab. S4). Among, a significantly negative correlation between the RRM1 expression and survival time was observed, and patients with high RRM1 expression exhibited a worse prognosis (Fig. 1C). Collectively, these results suggest that RRM1 may be a potential predictor to estimate patients’ survival.

**“Insert Figure S1 and Table S3-S4”**

**RRM1 promotes cell proliferation of human ESCC**

To study the biological function of RRM1 in ESCC, the levels of RRM1 mRNA in eight ESCC cell lines were firstly detected using real-time qPCR analysis (Fig. S2A). KYSE150 and KYSE410 cell lines with higher RRM1 mRNA were chosen for RRM1-knockdown experiment, and KYSE450 and TE-9 cell lines with lower RRM1 expression were used for RRM1 over-expression experiment. The efficiency in RRM1 knocked-down and over-expression cells were also confirmed by Western blot (Fig. S2B). Analysis of cell cycle by flow cytometry showed the S phase of both RRM1 knocked-down KYSE150 and KYSE410 cell lines significantly increased , but G2 phase significantly reduced, indicating that cell cycle progression was blocked in S/G2 phase (Fig. S3A). MTT assay (Fig. S3C) and colony formation assay (Fig. S3E) also confirmed that RRM1-silencing inhibited the cell proliferation. Moreover, the promoted cell cycle (Fig. S3B) and increased cell proliferation (Fig. S3D and F) and were observed in RRM1-overexpresing cells. Above results indicated that RRM1 acts as a cancer-promoting gene in ESCC.

**“Insert Figure S2 and S3”**

**RRM1 blocks glucose catabolism, but increases antioxidant capacity**

Based on the targeted metabonomics and analysis and metabolite set enrichment analysis method, a total of 139 metabolites in RRM1-overexpresing cells was identified. 2'-Deoxyguanosine 5'-monophosphate (dGMP), deoxycytidine monophosphate (dAMP), deoxyadenosine monophosphate (dCMP), and deoxyinosine was significantly increased (Fig. S4). Moreover, increased intermediate metabolites of glycolysis including D-glucose 6-phosphate (G6P), phosphoenolpyruvat (PEP), reduced nicotinamide adenine dinucleotide (NADH), and adenosine triphosphate (ATP) was also observed, but the levels of L-Lactic acid and malic acid involved in the tricarboxylic acid cycle were reduced, indicating a blocked glucose catabolism in RRM1-overexpresing cells. The pentose phosphate pathway (PPP) and glutathione system closely related with nucleic acid anabolism and intracellular antioxidant capacity was enhanced, and intermediate metabolites in both processes including nicotinamide adenine dinucleotide phosphate (NADP), D-ribose 5-phosphate (R5P) and glutathione disulfide (GSSG) were significantly up-regulated. Further tests confirmed the both enhanced process, significant up-regulated NADP, NADPH, GSH and GSSG in RRM1 over-expressing cells were noted as showed in Fig. S5A-D. The enhanced intracellular antioxidant system resulted in the reduced oxidative stress which was cleared with reduced reactive oxygen species (ROS, Fig. S5E). Lower ROS caused the down-regulation of three key tumor suppressor proteins, phos-p38, phos-p53 and p21 (Fig. S5F), which increased the phosphorylation of Rb and finally promoted cell cycles.

**“Insert Figure S4 and S5”**

**RRM1 blocks glycolysis process by participating in the phosphorylation of PKM2**

To identify the target proteins regulated by RRM1, protein mass spectrographic analysis was performed in RRM1 over-expresing cells. Pyruvate kinase M2 (PKM2) was found be the potential protein interacted with RRM1 by protein mass spectrographic analysis, and immunocoprecipitation cleared the interaction relationship between both proteins (Fig. 1E and F). In glycolytic pathway, pyruvate kinase (PK) is a key enzyme that catalyzes the transfer of a phosphoryl group from phosphoenolpyruvateto to ADP to form pyruvate and ATP, and the activity of PK is closely related with the phosphorylation of PKM2. The up-regulated phosphorylation levels of PKM2 were observed in both RRM1-expresing cells (Fig. 1G and H), and the inhibited activity of PK and reduced PA levels were also found (Fig. 1I and J), indicating that hyperphosphorylation of PKM2 might be the main reason causing the decreased PK activity. The immunohistochemical analysis also revealed a significant positive correlation between the expression levels of RRM1 and p-PKM2 (Fig. 1D).

**Treatment of gemcitabine and TEPP-46 reduces cancer cell viability**

Carcinogenesis of RRM1 may work by controlling the phosphorylation of PKM2 in human ESCC. To further clarify the regulatory mechanism, a inhibitor (gemcitabine) of RRM1 activity was used in RRM1-overexpressing cells. Results showed that the PKM2 phosphorylation level significantly decreased compared to that of control groups (Fig. S6A), but the PK activity and PA levels significantly increased (Fig. S6C and E). The enhanced PK activity promoted more intermediates into tricarboxylic acid cycle, and reduced the carbon flux to PPP, inhibiting the synthesis of antioxidants and re-increasing the oxidative stress levels (Fig. S6G). Increased oxidative stress resulted in the slowed cell cycle progression, and less deoxyribonucleotide caused by reduced RRM1 activity inhibited DNA production, finally slowing-down cancer cell proliferation (Fig. S6I). Similar results have been achieved by applied activator of PKM2 activity (TEPP-46) in RRM1-overexpressing cells(Fig. S6B, D, F, H and J), indicating a better clinical efficacy in treatment of gemcitabine and TEPP-46 on esophageal squamous cell carcinoma.

**“Insert Figure S6”**

**Table S1.** The clinicopathological characteristics of 105 ESCC patients used for immunohistochemistry analysis.

| **Parameters** |  | **Number of cases** | **Percentage (%)** |
| --- | --- | --- | --- |
| **Age (years)** | < 60 | 41 | 60.95 |
|  | ≥ 60 | 64 | 52.38 |
| **Gender** | Male | 55 | 47.62 |
|  | Female | 50 | 2.86 |
| **Pathological grade** | G1 | 3 | 93.33 |
|  | G2 | 98 | 3.81 |
|  | G3 | 4 | 25.71 |
| **Smoking history** | Yes | 27 | 74.29 |
|  | No | 78 | 16.19 |
| **Drinking history** | Yes | 17 | 83.81 |
|  | No | 88 | 1.90 |
| **T classification** | T1 | 2 | 25.71 |
|  | T2 | 27 | 17.14 |
|  | T3 | 18 | 55.24 |
|  | T4 | 58 | 60.95 |
| **N classification** | N0 | 64 | 26.67 |
|  | N1 | 28 | 12.38 |
|  | N2 | 13 | 98.10 |
| **M classification** | M0 | 103 | 1.90 |
|  | M1 | 2 | 23.81 |
| **Clinical stage** | I | 25 | 35.24 |
|  | II | 37 | 39.05 |
|  | III | 41 | 1.90 |
|  | IV | 2 | 60.95 |

**Table S2.** Specific primer pairs for the RRM1, p38, p53, CDKN1A, Rb and GAPDH genes used in quantitative real-time polymerase chain reaction analysis.

| **Genes name** | **Specific primers (5'to3')** |
| --- | --- |
| RRM1 | GCCGCCAAGAACGAGTCAT^a^ |
|  | AGCAGCCAAAGTATCTAGTTCCA^b^ |
| p38α | CCCGAGCGTTACCAGAACC^a^ |
|  | TCGCATGAATGATGGACTGAAAT^b^ |
| p53 | CAGCACATGACGGAGGTTGT^a^ |
|  | TCATCCAAATACTCCACACGC^b^ |
| CDKN1A | TGTCCGTCAGAACCCATGC^a^ |
|  | AAAGTCGAAGTTCCATCGCTC^b^ |
| Rb | CTCTCGTCAGGCTTGAGTTTG^a^ |
|  | GACATCTCATCTAGGTCAACTGC^b^ |
| GAPDH | TGTGGGCATCAATGGATTTGG^a^ |
|  | ACACCATGTATTCCGGGTCAAT^b^ |

^a^ Forward primer. ^b^Reverse primer.

**Table S3.** Relationship between RRM1 expression and clinicopathological characteristics of ESCC patients was tested by chi-square test.

| **Parameters** | **Number of cases** | **RRM1 expression** | | **chi-square test** | |
| --- | --- | --- | --- | --- | --- |
|  |  | **Low** | **High** | **Χ^2^ values** | ***p* values** |
| **Overall** | 88 | 30 | 58 |  |  |
| **Age (years)** |  |  |  |  |  |
| < 60 | 34 | 10 | 24 | 0.540 | 0.462 |
| ≥ 60 | 54 | 20 | 34 |  |  |
| **Gender** |  |  |  |  |  |
| Male | 49 | 14 | 35 | 1.499 | 0.221 |
| Female | 39 | 16 | 23 |  |  |
| **Pathological grade** |  |  |  |  |  |
| G1 | 2 | 0 | 2 | 1.120 | 0.571 |
| G2 | 81 | 28 | 53 |  |  |
| G3 | 5 | 2 | 3 |  |  |
| **Smoking history** |  |  |  |  |  |
| Yes | 23 | 8 | 15 | 0.007 | 0.935 |
| No | 65 | 22 | 43 |  |  |
| **Drinking history** |  |  |  |  |  |
| Yes | 15 | 5 | 10 | 0.05 | 0.946 |
| No | 73 | 25 | 48 |  |  |
| **T classification** |  |  |  |  |  |
| T1 | 1 | 0 | 1 | 7.988 | 0.046* |
| T2 | 24 | 9 | 15 |  |  |
| T3 | 12 | 0 | 12 |  |  |
| T4 | 51 | 21 | 30 |  |  |
| **M classification** |  |  |  |  |  |
| M0 | 87 | 30 | 57 | 0.532 | 0.469 |
| M1 | 1 | 0 | 1 |  |  |
| **N classification** |  |  |  |  |  |
| N0 | 56 | 17 | 39 | 3.042 | 0.219 |
| N1 | 20 | 10 | 10 |  |  |
| N2 | 12 | 3 | 9 |  |  |
| **AJCC stage** |  |  |  |  |  |
| I + II | 53 | 17 | 36 | 0.241 | 0.624 |
| III + IV | 35 | 13 | 22 |  |  |

**p* < 0.05

**Table S4.** Univariate and multivariate analysis for overall survival

| **Parameters** | **Univariate analysis** | | | **Multivariate analysis** | | |
| --- | --- | --- | --- | --- | --- | --- |
|  | **HR** | **95% CI** | ***p*** | **HR** | **95% CI** | ***p*** |
| **RRM1 expression**  **(Low vs High)** | 2.525 | 1.271-5.019 | 0.008* | 3.270 | 1.545-6.920 | 0.002* |
| **Age**  **(< 60 vs ≥ 60)** | 0.835 | 0.470-1.483 | 0.539 | 1.005 | 0.533-1.896 | 0.988 |
| **Gender**  **(Male vs Female)** | 0.958 | 0.537-1.709 | 0.885 | 1.045 | 0.554-1.971 | 0.891 |
| **Smoking History**  **(Yes vs No)** | 1.646 | 0.796-3.408 | 0.179 | 2.263 | 0.537-9.541 | 0.266 |
| **Drinking History**  **(Yes vs No)** | 1.375 | 0.613-3.083 | 0.439 | 0.664 | 0.132-3.346 | 0.620 |
| **T classification**  **(T1+2 vs T3+4)** | 1.502 | 0.826-2.731 | 0.182 | 1.732 | 0.833-3.601 | 0.141 |
| **N classification**  **(N0 vs N1+2)** | 1.284 | 0.685-2.408 | 0.436 | 0.952 | 0.443-2.049 | 0.900 |
| **AJCC** **stage**  **(I+II vs III+IV)** | 1.997 | 1.123-3.550 | 0.018* | 2.191 | 1.192-4.024 | 0.011* |

**p* < 0.05
